# Notes and Comments

**Published:** 1922-03

**Authors:** 


THE
### HOSPITAL and HEALTH
### REVIEW

### NOTES AND COMMENTS.

### HEALTH AND THE GEDDES "AXE."

WHEN the public health services were formed under a central department in 1848 there were many critics. These still remain, despite the increasing proofs that it is truest economy for a nation to try and prevent disease. The formation of the Ministry of Health in 1919 as the new central authority for the purpose of supervising the health of the people as a whole was a signal for the opponents of State action to break out in a fresh chorus of complaint on the score of waste of public money.

The report of the five business men—four of whom, by the way, are Scotsmen—upon the expenditure of the Ministry of Health on public health services constitutes a complete reply to these critics. It is also a most impressive tribute to the wise direction of this country's central health organisation. The report speaks of the health services as being "undeniably beneficial." The same sums as last year, without any reduction whatsoever, are provided for the treatment of tuberculosis, for maternity and child welfare, for the treatment of venereal diseases, and for the welfare of the blind. The sum of £50,000 given as a grant from th Exchequer towards the expense of port sanitation will, however, cease, and the cost again be borne by the locality.

Sir George Newman has good reason to be satisfied with this investigation into the medical department of the Ministry of Health, especially as the Geddes Committee express the hope that falling prices and the greatly increased incentive to economy will assist the authorities responsible for public health services "still further to increase their beneficial work."

The report inevitably hits hard at the uneconomic housing scheme adopted by Mr. Lloyd George after the Armistice. The decision to limit the liability of local authorities to the produce of a penny rate immediately withdrew any incentive to economy. Even to-day there is no financial inducement to a local authority either to sell or in any way to reduce the burden of £55 per annum falling on the taxpayer in respect of each of the 176,000 houses to be built. This housing policy has been the mill-stone round the neck of the newly-born Health Ministry. All must regret that it should be thus handicapped in its infancy. An unsound system of State socialism has overloaded the machinery for central health administration. That housing scheme is now abandoned, and the health department should have freer play and a fairer chance.

One recommendation of the Geddes Committee is bound to have a far-reaching effect upon public health. The report asserts that the system that has grown up since 1913 of giving percentage grants, varying from 20 per cent. to 75 per cent., has been a money-spending device, has increased bureaucratic control, and has left the decision as to the amount to be spent not to the Government of the House of Commons, but to the local authorities. Accordingly it recommends that both for education and other social services a fixed grant, or a grant based on some definite unit, shall be paid.

This step will increase the incentive to local authorities to economise; will restrict the growth of the demands on the taxpayer; and should enable staff economies to be effected at both the Ministry of Health and at the Board of Education. With regard to the former, however, in view of the absurd charges made in certain quarters as to the "squandermania" of the Health Ministry, it is worthy of notice that the report asserts: "We have had submitted to us a very complete account of the administrative system of the Ministry and are satisfied that vigorous steps have been taken to reduce expenditure on staff."

The Geddes report is in fact another mile-stone on the road of national health. We have always recognised that the Ministry can only reform the machinery of government, and cannot be a substitute either for medical science or for the medical practitioner who is the instrument and interpreter of the science of medicine. But the fact that the national expenditure on public health services, except for port sanitation, are not to be reduced during the coming financial year by even one halfpenny, shows that, in the opinion of five shrewd business men, it is the truest economy to spend money to prevent ill-health.

---

### EARLY TREATMENT OF MENTAL DISEASE.

IN the annual report of the Crichton Royal Institution, Dumfries, Dr. C. C. Easterbrook, Physician Superintendent, calls attention to a grave defect in our lunacy laws, namely, the want of statutory provision for the treatment of early and mild cases of mental illness among the rate-aided class as voluntary patients in our mental hospitals, with their highly organised equipment and facilities, and their specially trained medical and nursing staffs. As the law stands at present, only private or "paying" patients can be admitted for treatment in mental hospitals as voluntaries. That this provision is appreciated by this class is indicated by experience at Crichton Royal for many years. In 1921 60 per cent. of the private patients admitted came voluntarily. The public or rate-aided patient, on the contrary, cannot be admitted into a mental hospital until his illness has advanced to the stage when it becomes possible for him to be certified as a "lunatic," or "insane person," or "person of unsound mind," and as a "proper person to be detained under care and treatment." Further, the procedure for his admission has to be effected through the agency of the Parish Council—which shares financial responsibility with the District Board of Control—as the result of which the patient also becomes, technically at least, a "pauper," and is described as such in the admission papers. This short-sighted policy in the treatment of those who constitute the great majority of the population in the public or rate-aided asylums of the country, by depriving the patients of the facilities for cure during the earlier and more hopeful stages of the disease, entails much permanent incurability and mental invalidism, with corresponding loss of efficiency to the community, that might be obviated under a more enlightened and, clearly, a more economical system. This statement is strongly supported by experience at Dumfries in the past four years, during which period the recovery rate of the voluntaries exceeded that of the certificated by approximately 10 per cent.

The present policy of denying access of rate-aided patients to mental hospitals as voluntaries has other adverse effects. It deprives the public asylums themselves, as hospitals of healing, of their fair and legitimate share of that healthy, curative atmosphere which an active circulation of hopeful and curable cases so largely promotes. Again, the existing system of compulsory pauperisation and certification, with no alternative, is largely responsible for the frequent unwillingness of the relatives to send the patient to a mental hospital until this step is absolutely necessary, and also for the creation in certain cases of superadded and undeserved feelings of resentment, shame and loss of self-respect, feelings which are not conducive towards recovery, and do not arise in the case of other statutory invalids, such as those treated in fever hospitals or in sanatoria for tuberculosis. The remedy is obvious. The invalid of the rate-aided class who requires treatment in a mental hospital should, like the private patient, be given the opportunity of going of his own accord. A simple statutory provision to this effect is long overdue. It would extend to the rate-aided class a boon which has already been much appreciated by private patients seeking treatment in those mental hospitals in Scotland which specially provide for this class.

---

### SCHOOL MEDICAL WORK.

AT the Leeds Division of the British Medical Association Dr. Wear read an interesting paper on School Medical Work. The first country to make a start in this direction, he said, was Sweden, in 1830, and by 1868 all Swedish schools were medically inspected. While the idea was gradually taken up by other countries, England did not really wake up to the meaning of school medical inspection until about 1905. The chief cause which led to the adoption of a scheme by this country was a Royal Commission on physical training in Scotland in 1903, which showed that deplorable conditions existed among school children. The School Boards of London and Bradford took action in 1891 and 1893 respectively, and by 1905 school medical officers were appointed by seventy-nine different local authorities. The Education (Administrative Provisions) Act of 1907 made it the duty of all authorities to provide medical inspection. Since that date many other Acts have been passed affecting the school medical service, practically the whole of which was consolidated into the Education Act of 1921.

School medical inspection in Leeds is carried out by eight full-time and three part-time medical officers, six dentists, 27 nurses, 24 clerks, and several dental attendants. In school medical work the main object is to make the school children cleaner and healthier, and by that means to raise the standard of health of the coming generation. The examination is comparatively simple, and occupies about seven minutes, or twenty per service. There are 120 schools in the city and 85,000 children on the roll, and three routine examinations are carried out for every child—on entering school, on leaving school, and when eight years of age. Teachers may bring forward any child for examination, and all children found defective have to be re-examined, usually six weeks after the routine examination. The dentists periodically visit each school, and enter treatment on record cards. Nurses visit all schools twice a year and examine all children as to cleanliness. Parents present at medical inspections are personally informed, and subsequently get a stereotyped notice: "Your child has been found to be suffering from so-and-so, and you are advised to obtain medical advice."

For administration purposes the city of Leeds is divided into six districts, each of which contains an approximately equal number of schools and children, together with a clinic. Each district is in charge of a medical officer with a number of nurses, the nurses assisting and carrying out minor treatment. The medical officer does all the examinations and re-examinations in his own schools, and attends his clinic two or three afternoons a week. The clinics vary in size; the largest has 57,000 attendances a year and the smallest 16,000. The total attendances for all purposes at all the clinics for the year 1920 was about 190,000. The minor ailments treated are discharging ears, blepharitis, impetigo and other skin diseases, and verminous heads and bodies. Eye work, including refraction and provision of spectacles, and massage and dental work, are also carried out. Before the days of clinics such work probably remained undone or was very badly done at home. None of this treatment is beyond the scope of the school clinic, as none of it would be undertaken by medical men, and would still be carried out in the home as of old without necessary supervision. In addition to the six clinics noted, there is also a seventh clinic, situated centrally, at which special cases of all kinds attend for more thorough examination, such as mentally defectives, blind, deaf, epileptic, cripples, and tuberculous cases. Here there are various departments—eye, nose, throat, dental, massage, and X-ray treatment for ringworm. At this clinic there were 19,000 attendances in 1920. Dental work is carried out at four clinics, and all extractions are under gas anæsthesia.

The aim of the school medical work is mainly preventive, the parent being advised to take the child to a medical practitioner for treatment. The school medical officers want the fullest help and the trust of the general practitioner.

### THROAT AND EAR DISEASE IN SCHOOL CHILDREN.

AN address delivered by Mr. T. B. Layton, Surgeon in charge of the Throat and Ear Department of Guy's Hospital, to members of the Invalid Children's Aid Association and of the Care Committees of L.C.C. Schools, and reported in the "Lancet," is of interest in view of the current controversy on the necessity of operations for enlarged tonsils and adenoids. He believes that far too many operations are done upon tonsils and adenoids in children, especially in the public work of hospitals and school clinics, whether they are done by general practitioners or by general surgeons, even more than when done by specialists. The idea that the lymphoid tissue of the pharynx is a useless structure which can be removed on mere suspicion without doing harm is wrong. The tonsils form a part of the first line of defence to the entrance of organisms into the body which produce disease. When they are inflamed we should not regard them as diseased, but as engaged in the process of defence. Mr. Layton regards a tonsil as diseased only when organisms are living and developing in it, so that they or the poisons they form can either extend out into the throat and cause trouble there, or by being absorbed into the body give rise to some general disturbance. This is what is meant when we say that the tonsils are a source of sepsis. One often hears that a child has frequent colds and that when he gets one it always flies to the throat. This child may be suffering from a cold caught by inspiration of organisms, and the inflammation may have extended down from the nose to the throat, or the child may be falling time and again a prey to its own microbes living in the tonsils. In the latter case a careful history will reveal that the sore throat comes first and the ill-feeling later; in the former the reverse is the case, and then attention to hygiene is more likely to benefit, whilst in the latter the tonsils may have to be removed.

A tonsil should never be removed, he holds, because it is enlarged, but only because it is diseased. Its enlargement is a sign that it is performing its normal function—the destruction of microbes. Before removing it the question has to be decided whether the harm done to the body by the disease is greater than the good done by the enlargement. Removal for mere enlargement Mr. Layton considers the most fertile cause of excess of operations to-day. On the other hand, nothing is more beneficial than the removal of tonsils and adenoids in properly selected cases. Operation must not be regarded as a panacea; neither must the medical treatment which is vaunted as a substitute for it. Some of the children suffering with symptoms of lymphoid enlargement of the naso-pharynx are suffering from a general disease or a general lowering of health, and others are suffering from a local disease. It is as wrong to treat all by general methods as it is to submit them all to operation.

Mr. Layton concluded with some good advice to health visitors. One of their duties is to persuade the parents to take the doctor's advice. When this is moderate and based upon due consideration they will seldom have any difficulty. When they have difficulty they should not try to do too much at once. Under the Children Act it is possible to summons parents for refusal to have an operation done. But it must be sparingly used. For the ultimate upraising of the health of the Nation the Ministry of Education will prove more important than the Ministry of Health. Health visitors' work lies where the two meet, and has an importance which is only beginning to be recognised.

### £750,000 FOR HOSPITALS.

THE February number was hardly in the hands of our readers when the extent to which King Edward's Hospital Fund will benefit under the late Lord Mount Stephen's will was made known. The residuary estate, bequeathed to the Fund, is expected to realise £750,000. A large capital sum would always be welcome, but it is doubly welcome to-day. In the first place, it replaces three times over the emergency grant bestowed recently by the Fund to the London hospitals, and, secondly, while increasing the total capital and consequent income of the Fund, it will enable the Committee of Management to take a large view of present necessities both before, and at the time of, the next allotment of grants. In these days of desperate straits the Fund, thus relieved, becomes more than ever a stabilising influence, and one cannot be sufficiently grateful to millionaires of public spirit like the late Lord Mount Stephen for securing, so far as their power lay, the foundations of the voluntary system. But we cannot rejoice in this bequest without remembering the heavy toll taken by the State from such bequests to charity at the very time when it is appropriating half a million to relieve them ! The irony is so patent that an occasion is given for once more raising the question of exempting bequests to charity as soon as Parliament meets. We hope that Members of both Houses will not lose the opportunity, and that the central agencies, including the Fund itself, will do their best to revive this discussion.

### MENTAL HOSPITALS.

ALL those who recognise that improvements are essential in the conditions under which the insane are treated in our mental hospitals are glad to see that the Departmental Committee appointed by Sir Alfred Mond to inquire and report upon charges made against asylum administration by Dr. Montague Lomax in his book " The Experiences of an Asylum Doctor " has commenced its meetings. For a time it appeared that the unfortunate refusal of Dr. Lomax to give evidence and the unwise decision of the Asylum Workers' Union not to appear, might mean the breakdown of the Committee. The objections taken by Dr. Lomax and others to the constitution of this Committee were obviously absurd, for the whole question of the mental hospitals depends on legal and technical considerations. No man without many years' experience of the question could hope to give decisions on such a problem. The meetings are to be open to the public, who will undoubtedly gain by viewing the question without believing too credulously the exaggerated statements that have recently been made on the subject.

### THE INFLUENZA EPIDEMIC.

THOUGH no large part of the country has escaped, London, South Wales and the North of England, with several great Scottish cities, seem to have been the districts hardest hit by the present—we may almost say, the recent—epidemic of influenza. The pneumonic type of the disease has not been common and has been confined mainly to elderly people. The outbreak has not approached that of 1918-19 in severity, but in many districts it has been severe enough to strain local resources to the utmost, and even mild cases may be followed by that prolonged depression and lowered vitality which reduce healthy people to the level of miserable hypochondriacs. A noticeable feature of the epidemic has been the comparative readiness of the victims to take to their beds while they are still on the right side of collapse, a gain which is possibly due to vivid memories of 1918-19. One almost hopes that the person who used to boast that he had not " given in," but continued to work with the disease upon him, is literally extinct. Glances of hatred are cast at the cougher or sneezer in public vehicles, a wide berth is given to him in the office ; public opinion condemns what it once admired.

### ECONOMY AND MATERNITY AND CHILD-WELFARE SERVICES.

MANY local authorities in Scotland are protesting vigorously against the use of the economy axe on maternity and child-welfare services. Being one of the most recent health services to develop, it is perhaps suffering most. Although much useful work was done before and during the war, it has been since the Armistice that many local authorities, after much propaganda by the Government and others interested in public health, have produced schemes for approval. The production of the schemes has in many cases coincided with the Government's decision to economise on these services, and the Department that did so much to stimulate enthusiasm for maternity and child-welfare work is now faced with the unpleasant duty of repulsing that enthusiasm. The child-welfare movement in Scotland has done specially good work. The local authorities that have had their schemes in operation are anxious to develop them and, at the very least, to keep them up to the present state of efficiency ; the local authorities which have not yet schemes in operation have been convinced by the manifestly excellent results achieved by others, and do not see why they, too, should not be allowed to do as other local authorities are doing. There is a strong public opinion in Scotland in favour of the development of the child-welfare movement, and there are few economies on the social services that will be more unpopular in Scotland.

### INFANT WELFARE INSTITUTIONS.

THE list just issued of the institutions maintained for expectant and nursing mothers and children under 5 in England shows how seriously infant welfare has been affected by the present shortage of money. There are now only 104 homes and hospitals for mothers with their babies as against 116 in 1920. This means that 100 fewer beds are available. The societies which are mainly responsible for these institutions are most of them in desperate financial straits. The homes for children under 5 have decreased by an even greater number, and there are roughly 800 beds instead of 1,000. A study of the list of these institutions shows, hoewver, that the number of maternity homes has increased from 88 to 123 and the beds in such institutions from 1,100 to 1,600.

### DENTAL COLLEGE FOR GLASGOW.

DR. W. G. DUN, President of the Royal Faculty of Physicians and Surgeons, in his recent address to the Glasgow Odontological Society, urged the need for a new Dental Hospital and College for Glasgow. He judged that the dental colleges in the United States might be their model. The photographs which he had seen of these institutions impressed him with both the buildings and their equipment. He thought that the Act of 1921 would end unqualified dental practice. The unqualified man, of several years' experience, was provided for in Section 3, which permitted *bona-fide* dental mechanics to be registered, provided that they passed the prescribed examination before July 28, 1931; persons who had been practising for less than five years might be registered if they passed before July 28, 1923. The examination would be fairly stiff. Mr. J. A. Young, president of the Society, said that the dental profession would be much increased. But he hoped that their societies would welcome the new members and try to infuse the professional spirit into them. This judicious counsel is enforced by the belief that the door will now be closed to improper practice.

### HOSPITAL SALARIES AND WAR BONUSES.

THE adjustment of hospital salaries to the fall of current prices, which generally means the reduction of the war bonus, has placed the committee of the Skipton Infectious Diseases Hospital in some difficulty. While the bonus was added when prices were high, and therefore apparently justly reducible, it is urged that the salaries paid at Skipton are lower than in other institutions of its class. The reductions are therefore being postponed pending an inquiry. This is more than a mere instance. It is hardly denied that before the war the salaries paid to hospital officials were lower than such men could have commanded for similar work in other walks of life. The low salaries of nurses were notorious. Thus the reduction of war bonuses in hospitals is an exceptionally delicate matter. Happily, under the voluntary system, disputes very rarely arise; but what we still need are standard salaries and standard pensions. An article on this subject appears on page 164.

### LEEDS AND THE VOLUNTARY HOSPITALS COMMISSION.

THE deputation from the hospitals of Leeds which has waited on Lord Onslow, Chairman of the Voluntary Hospitals Commission, to urge that the whole of Yorkshire should be placed under one Local Committee, is understood to have received a satisfactory reply. If the North Riding will agree to the Leeds proposal, the plan will probably go through. This is interesting because it is an early instance of the readiness of the Voluntary Hospitals Commission to meet the demands of local hospitals, so far as these can be fulfilled without affecting the conditions already laid down by the Commission. We trust that this sympathetic attitude in an early plea will not be lost on those localities affecting suspicion of the Commission and a desire to stand aside from the Local Committees which it is setting up. Since, in his reply to the Leeds deputation, Lord Onslow is reported to have said that the size of the area under a Local Committee was not prescribed by the conditions imposed, those districts willing to co-operate may be able to bring pressure to bear upon neighbouring and recalcitrant areas. The very opposition shown in places to the Local Committees proves, we think, the need for them. Hospital co-ordination must come from above.

### LANCASHIRE LOCAL COMMITTEES.

THE first meeting of the Local Committee for the County of Lancashire, excluding the chief towns, under the Voluntary Hospitals Commission, was noteworthy for a suggestion that a Joint Advisory Committee should be formed by all the Local Committees in the county. The proposed advisory committee, it was proposed, should consider whether three Local Committees in Lancashire were desirable. This, at all events, is a sign that the new committees are beginning to think in areas, and, if their proposals lead the hospitals to do so also, a real step toward a common policy will have been taken. Mr. Walter Davies is chairman of the Manchester Local Committee, and Mr. J. J. Cockshott, chairman of the County Committee.

### EDINBURGH ROYAL INFIRMARY AND THE APPROVED SOCIETIES.

A CURIOUS explanation has been lately offered for the decision of the approved societies not to make grants to the Edinburgh Royal Infirmary. It is stated that this hospital is alone among local institutions in declining to supply medical certificates to patients who are members of approved societies, in order that they may receive benefits, except when a fee of one guinea is paid. Apparently, however, the infirmary will grant a certificate, on the application of the local secretary, after such a patient has been discharged. If the infirmary is indeed alone in this, an explanation is certainly desirable. For, while it is true that many demands are made upon hospitals and their staffs, such as the giving of evidence in coroners' courts, for example, which are not, and ought to be, paid for, it is also true that a hospital should not be charged with exceptional conduct in a relatively minor matter of this kind. Mr. W. S. Caw, O.B.E., the treasurer and clerk, must be as alive as any man to the mutual advantages of co-operation between hospitals and approved societies. Perhaps he will either explode a fallacy or supply the official justification of the practice attributed to his committee?

### A KEEN LOCAL COMMITTEE.

THE Somerset Voluntary Hospitals Committee is going very carefully to work, and has decided to meet monthly in the different hospitals of the county, so that all may become personally known to the members. The meetings will also be used to collect the information necessary for a survey of the hospital position throughout the county, in order that defects in the present hospital service may be discovered, and a policy determined for the raising of the necessary funds. The Committee has addressed a series of questions to each hospital committee and staff; and similar questions are being also sent to the panel committees and to a certain number of medical men in the City of Bath. The answers to these questions are expected not only to contain information but to elicit suggestions; and whatever be the conclusions arrived at, they will be the result of comprehensive study.

### A NEW REGIONAL COMMITTEE.

ON the initiative of the Sheffield Hospitals' Council, a conference of hospitals has been held to form a Regional Committee under the British Hospitals Association. We are always glad to see the number of the Association's Regional Committees increase, for there can be no doubt that this body took on a new lease of usefulness when it was thus decentralised. There is another reason, perhaps, why these regional committees may be useful at present. They are composed, of course, of hospital representatives, and should any misunderstanding arise between any hospital and the new Local Committees formed under the Hospitals' Commission, the Regional Committee, though its area is not necessarily the same as that which the Local Committee represents, may be able to express hospital opinion better than any isolated hospital can do. There thus exist two bodies, the new Local Committee, designedly not consisting of hospital men, and the Regional Committee of the British Hospital Association, representing wholly institutional opinion. The Local Committees are more numerous, but their existence and that of the regional committees should, from two different angles, help the hospitals to think more in areas and encourage the co-ordination at present lacking. We hope that they will stimulate each other, for there is no reason why they should get in one another's way.

### FEVER NURSING AS A SPECIAL CAREER.

THE difficulty of securing fever nurses was discussed at a recent meeting of the Ealing and Chiswick Isolation and Maternity Hospitals Committee, when, in reply to questions, Dr. Orr explained the facts of the case. We pay, he said, the usual rates of salary laid down by the Fever Nurses' Association. The difficulty of getting fever nurses is that training in a fever hospital does not count for general training. That is true and the moral seems to be that fever nursing must be made as attractive a career as that of general nursing. Hitherto fever nursing and asylum nursing have laboured under many defects, and consequently have been often recruited by those who regarded them as stop-gaps and did not intend or could not afford to remain in them. Fever nursing in the past has not always been recruited from the most promising type of probationer. The salaries and status are being improved, but the difficulties will remain until fever nursing is made a special career as attractive at least as its sister branches.

### THE M.O.H. AND THE ISOLATION HOSPITAL.

SHOULD medical officers of health who are asked to act as consultants to Isolation Hospitals be paid a fee for each consultation, or receive a fixed payment for the work of the position ? Dr. E. H. T. Nash, the medical officer of health for Heston and Isleworth, holds the latter view, as a matter of principle. He contends that medical superintendents, anxious to show the economy of their administration, hesitate to incur the expense of consultations, if they are paid for on each occasion, and that, if a fee is paid at all, it should be a fixed sum, regarded as an insurance in the interests of the patients. Since he offered to do the work proposed for nothing, if the Isolation Hospital Committee desired this, his ground is unquestionably sincere, and it is also probable that a fixed fee secures both economy and the best interests of the patients. This committee, however, refused to look at the matter in this light, or even to accept the service voluntarily offered. An honorarium rather than a salary would, however, have met the difficulty, and in the absence of a reasoned reply, Dr. Nash's arguments stand as they did.

### MONOTONOUS MENUS.

WE were glad to see that a visiting Commissioner of the Board of Control lately urged the authorities of a provincial mental hospital to take the first opportunity to vary the monotony of the breakfasts and teas. These apparently are confined at present to bread and margarine for breakfast, and bread and margarine for tea. The need for economy is potent, but it must be a reflection on any institutional cook who, if allowed to do so, cannot vary such a diet out of existing resources. Even bread and dripping would be no negligible change ; and could not the bakehouse produce something with its flour that shall have a different flavour from bread ? The variety of scones is innumerable. At present, institutional cooks are often little more than mechanics for roasting joints in gas ovens and changing shins of beef into beef tea. But we hear in America of hospital dietitians and at home of hospital housekeepers. One of the tests of the latter should be the absence of a fixed monotony of diet.

### RED CROSS DEPOTS FOR HIRE OF SICK-ROOM APPLIANCES.

WE have already described the Red Cross plan to establish depots where the needs of the sick at home can be supplied on hire with the utensils and appliances usually required in sickness and convalescence. The Priory for Wales of the Order of St. John has now formed six depots in Cardiff, not to mention others in different towns of South Wales. The Carnarvonshire County Nursing Committee has already thanked the Priory for this new departure, and once they get known, these depots will be appreciated by many medical men who can now tell their patients, where professionally recommended, of centres from which everything from a bed-pan to a bath-chair can be hired at a nominal cost. The purchase of such articles has always added to the cost of illness extravagantly, since they are usually wanted only for a short time. The conditions of hire, under the new scheme, are simple and reasonable. In South Wales the equipment has sometimes been provided by the workmen's generosity.

